# Microbial bionic nano-aromatic drugs for prevention of depression induced by chronic stress

**DOI:** 10.1186/s12951-024-02382-y

**Published:** 2024-04-12

**Authors:** Ruiyuan Liu, Tianlu Zhang, Chaobo Bai, Jing Chen, Xin Zhang, Guiying Liu, Songjie Shen, Junliang Yuan, Zhiguo Lu

**Affiliations:** 1 College of Pharmacy, Heze University, Heze, 274015 PR China; 2 State Key Laboratory of Biochemical Engineering, Institute of Process Engineering, Chinese Academy of Sciences, Beijing, 100190 PR China; 3 Department of Neurology, Peking University Sixth Hospital, Peking University Institute of Mental Health, NHC Key Laboratory of Mental Health (Peking University), National Clinical Research Center for Mental Disorders (Peking University Sixth Hospital), Beijing, 100191 PR China; 4 Department of Breast Surgery, Peking Union Medical College, Peking Union Medical College Hospital, Chinese Academy of Medical Sciences, Beijing, 100730 PR China; 5 Department of Pediatrics, Capital Medical University Affiliated Beijing Anzhen Hospital, Beijing, 100029 PR China; 6 Key Laboratory of Biopharmaceutical Preparation and Delivery, Institute of Process Engineering, Chinese Academy of Sciences, Beijing, 100190 PR China

**Keywords:** Microbial bionic nano-aromatic drugs, Aromatherapy, Prevention of depression, Chronic stress, Sustain release of essential oils

## Abstract

Depression is a mood disorder mainly clinically characterized by significant and persistent low spirits. Chronic stress is the leading cause of depression. However, traditional medicine has severe side effects in treating depression, ineffective treatment, and easy recurrence. Therefore, it is of great significance to prevent depression in the environment of chronic stress. In this study, aromatherapy was used for the prevention of depression. To solve the defects of intense volatility and inconvenience in using essential oils, we designed bionic nano-aromatic drugs and adhered them to the wallpaper. Inspired by the moldy wallpaper, we successively prepared the morphology-bionic nano-aromatic drugs, the function-bionic nano-aromatic drugs, and the bionic plus nano-aromatic drugs by referring to the morphology of microorganisms and substances in bacterial biofilms. Bionic nano-aromatic drugs remarkably promoted their adhesion on wallpaper. Molecular dynamics simulation explored its molecular mechanism. The essential oils, which were slowly released from the bionic nano-aromatic drugs, showed excellent biosecurity and depression prevention. These sustainedly released essential oils could significantly increase monoamine neurotransmitters in the brain under a chronic stress environment and had excellent neuroprotection. Besides, the bionic nano-aromatic drugs with simple preparation process and low cost had excellent application potential.

**Supplementary Information:**

The online version contains supplementary material available at 10.1186/s12951-024-02382-y.

## Introduction

Depression is a common affective disorder characterized by marked and persistent depressed mood, accompanied by low sleep quality, decreased appetite, slow thinking, mental unfocused, lack of interest, and other symptoms [1–4]. The severe ones will tend to commit suicide [5, 6]. The pathogenesis of depression is related to genetic, physiological, and environmental factors [7–11]. Generally speaking, treatments for depression include medication, psychotherapy, and physical therapy. These treatments have a long cycle, high cost, severe side effects on the body, and relapse. By contrast, the prevention of depression can avoid depression and is applied to prevent the recurrence of depression after treatment.

Among environmental factors, stress is the leading cause of depression [12, 13]. Inadequate secretion of neurotransmitters such as serotonin is the primary physiological factor for depression [14, 15]. Therefore, relieving stress and increasing the release of neurotransmitters such as serotonin in the brain are expected to prevent depression effectively. Moreover, the prevention of depression is long-term. Therefore, preventing depression must be safe, low-cost, universal, does not affect life and work. Drug prevention is not desirable. Besides, doing some things and conducting regular psychological counseling will also bring a heavy burden.

Aromatherapy is a method of preventing and treating diseases using aromatic drugs, and it is increasingly used as an adjuvant treatment for mental diseases [16, 17]. Many studies have pointed out that lemon essential oil (LEO) and bergamot essential oil (BEO) can relieve stress [18–20]. The inhaled essential oils can transmit signals through the olfactory system to stimulate the brain to release various neurotransmitters, thus further regulating emotions. However, excessive volatilization of essential oils reduces the service life of essential oils and severely weakens the prevention of depression. Besides, regular aromatherapy to prevent depression will bring great inconvenience. Therefore, the key of aromatherapy to prevent depression is to slow the release rate of essential oils and improve convenience.

Nanobiotechnology is widely used to slow the release of molecules [21–27]. However, most nanomaterials slowly release drugs in the solution rather than in the atmosphere. Mesoporous silica nanoparticles (MSNs) have a large specific surface area and strong adsorption capacity for aromatic drug molecules, thus significantly slowing the release rate of the molecules [28–30]. Besides, compared with other nanomaterials, the preparation method of the MSNs is simple, and the cost is low. Adding essential oils to daily necessities, the convenience of aromatherapy can be improved, thus reducing the burden on work and life. Improving the adhesion of essential oils to daily necessities was the key to improve the convenience of aromatherapy.

**Fig. 1:**
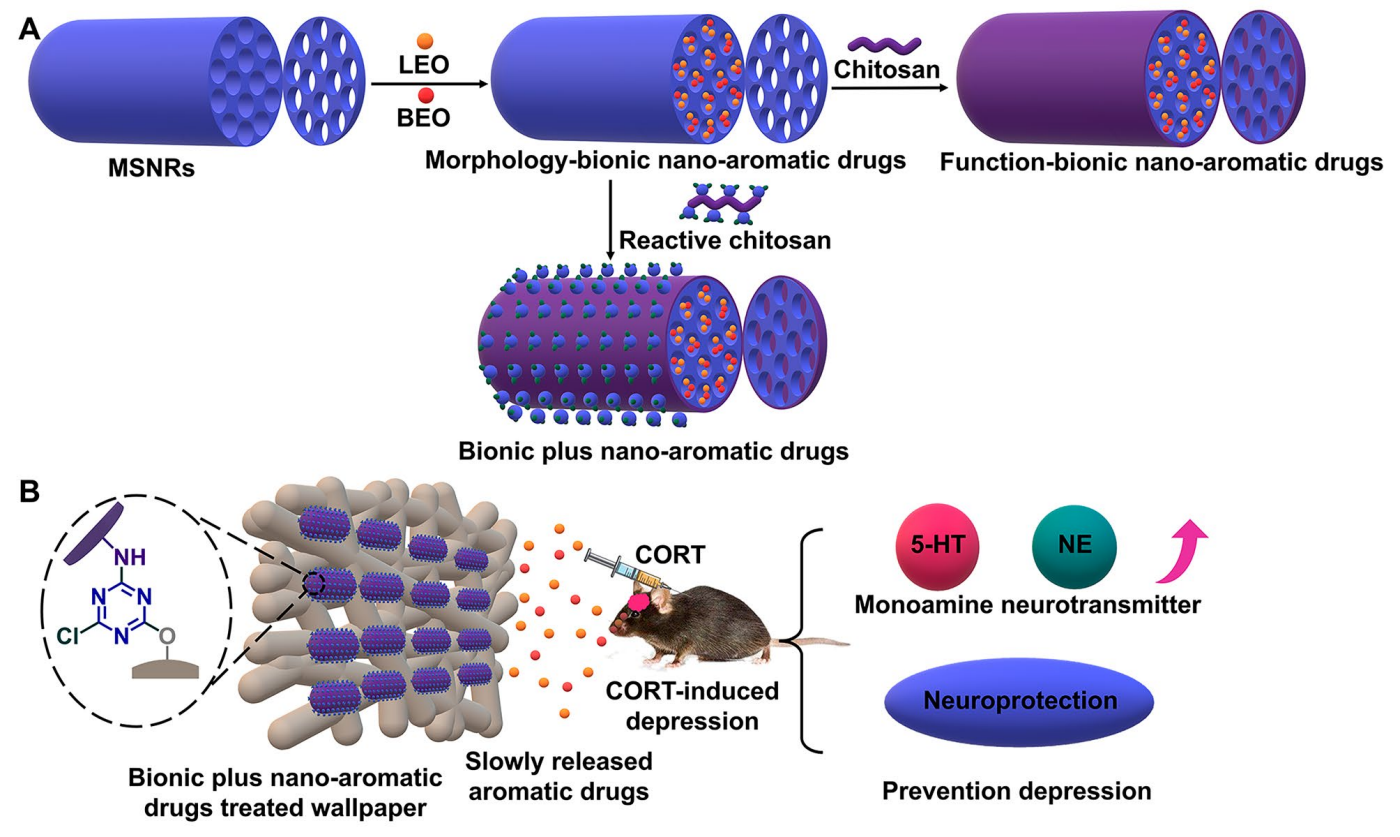
The schematic diagram. (**A**) The schematic diagram of biomimetic nano-aromatic drugs. (**B**) The schematic diagram of adhesion of bionic nano-aromatic drugs on wallpaper and preventing depression of bionic nano-aromatic drugs

In this study, we proposed a new concept of bionic nano-aromatic drugs inspired by the moldy wallpaper. First, many microorganisms have a rod-like morphology, increasing the contact area of the microorganisms with the substrate, thereby improving adhesion. Besides, polymeric substances in bacterial biofilms, especially polysaccharides, can improve the adhesion of microorganisms to substrates through hydrogen bonds, electrostatic forces, and Van der Waals forces [31]. However, microorganisms are unable to form covalent bonds with that substrate, and thus adhesion remains defective. We introduced the covalent bond based on the function-bionic and promoted the adhesion of nano-aromatic drugs on the wallpaper. As shown in Fig. 1A, we first synthesized the mesoporous silica nanorods (MSNRs). The LEO and BEO were adsorbed to form the morphology-bionic nano-aromatic drugs. Subsequently, the morphology-bionic nano-aromatic drugs’ surface was modified with chitosan to form the function-bionic nano-aromatic drugs. The cyanuric chloride (CYC) was modified on the chitosan to form reactive chitosan, modified on the morphology-bionic nano-aromatic drugs’ surface to form the bionic plus nano-aromatic drugs. The bionic plus nano-aromatic drugs with bionic and covalent interactions firmly adhered to the wallpaper (Fig. 1B). The severe stressful environment leading to depression was created by subcutaneous injection of corticosterone (CORT). Under this stressful environment, the wallpaper treated with bionic plus nano-aromatic drugs was pasted on the mouse cage to sustained release the essential oils. The essential oils relieved the stress of the mice. It increased the contents of neurotransmitters, such as serotonin (5-HT) and norepinephrine (NE), to regulate the mice’s emotions and prevent depression.

## Materials and methods

### Materials

Hexadecyltrimethylammonium bromide (CTAB, 99%), (3-aminopropyl)triethoxysilane (APTES, 98%), chitosan, superdry tetrahydrofuran (THF, 99.5%) and N,N-diisopropylethylamine (DIPEA, 99%) were purchased from J&K Chemical. Cyanuric chloride (CYC, 99%) was purchased from Energy Chemical. Bergamot oil and lemon oil were purchased from Shanghai Yuanye Bio-Technology Co.,Ltd. Ammonium hydroxide (AR, 25−28%), hydrogen chloride (HCl, AR, 36−38%), ethanol (AR), acetone (AR), dimethylsulfoxide (AR, DMSO), cyclohexane (AR), methanol (AR) and dichloromethane (DCM) were obtained from Sinopharm Chemical Reagent Co.,Ltd. CORT (97%) was purchased from TCI.

### Preparation of MSNRs

CTAB (680.4 mg) was dissolved in 140 mL of deionized water, and ammonia (4 mL) was added. Dissolve under ultrasound and stir for 30 min. TEOS (2.928 mL) was added dropwise with stirring and reacted at 40 ℃ for 2 h. After the reaction was completed, the precipitate was obtained by suction filtration under reduced pressure and washed three times with ethanol. The resulting precipitate was removed by vacuum drying. Subsequently, the templating agent CTAB was removed by extraction. Redisperse 1 g of the product in a mixture of ethanol and hydrochloric acid (100/1, v/v) to remove CTAB. The product was collected by centrifugation (4000 rpm) and washed with ethanol after three refluxes at 80 ℃ each for 24 h. The organic solvent was subsequently removed by vacuum drying. The morphology of the MSNRs was characterized by scanning electron microscopy (SEM), JSM-6700 F, and the internal structure was characterized by transmission electron microscopy (TEM, JEM-2100). Nitrogen adsorption-desorption isotherms determined the pore properties of the rod-like MSNs. The MSNs were heated to degassed condition at 180 °C for 8 h. The specific surface area, pore size, and pore volume were then measured and calculated using the Brunauer-Emmett-Teller (BET) method.

### Preparation of morphology-bionic nano-aromatic drugs

MSNRs (100 mg) were dissolved in a mixture of BEO (2 mL) and LEO (2 mL) and stirred at room temperature for 24 h. The precipitate was subsequently obtained by filtration under reduced pressure and washed three times with ethanol. Vacuum drying at 40 ℃ to remove the ethanol solvent on the surface of the nanoparticles, thereby obtaining the bionic nano aromatic drug with the appearance. The morphology of the morphology-bionic nano-aromatic drugs was characterized by SEM (JSM-6700 F), and the internal structure was characterized by TEM (JEM-2100). Nitrogen adsorption-desorption isotherms was employed to detect the pore properties of the bionic-shaped nano-aromatic drugs. The topography-mimicking nano-aromatic drug was heated to a degassed state at 180 °C for 8 h. The specific surface area, pore size, and pore volume were then measured and calculated using the Brunauer-Emmett-Teller (BET) method. Fourier transform infrared spectroscopy (FT-IR) was employed to characterize the chemical modification of the morphology-bionic nano-aromatic drugs. The encapsulation efficiency of the aromatic drug was measure by thermogravimetric analysis (TGA).

### Preparation of reactive chitosan

Chitosan (2 g), CYC (2 g), and DIPEA (3.3 g) were dissolved in THF (100 mL) and reacted with stirring at 5 °C for 24 h. At the end of the reaction, the precipitate was obtained by filtration under reduced pressure and washed three times with a mixture of methanol and THF (3/1, v/v). The precipitate was then placed in a vacuum drying oven to remove the organic reagents. FT-IR was employed to characterize the chemical structure of reactive chitosan.

### Preparation of function-bionic nano-aromatic drugs

Chitosan (60 mg) was dissolved in water with acetic acid (10%, v/v) and sonicated for 30 min to dissolve. The pH was adjusted to 6.0 with 1 M sodium hydroxide solution, and then the morphology-bionic nano-aromatic drugs (50 mg) were added. After stirring for 24 h, the precipitate was obtained by centrifugation (5000 rpm) and washed three times with deionized water. The precipitate was dried at 40 °C for 2 h to remove water molecules from the surface of the nanoparticles. The morphology of the function-bionic nano-aromatic drugs was characterized by SEM (JSM-6700 F), and the internal structure was characterized by TEM (JEM-2100). The chemical modification of the functional bionic nano aromatic drug was characterized using FT-IR. The encapsulation efficiency of the essential oils was measured by TGA.

### Preparation of bionic plus nano-aromatic drugs

Reactive chitosan (60 mg) was dissolved in deionized water with acetic acid (10%, v/v) and sonicated for 30 min. The pH was adjusted to 6.0 with 1 M sodium hydroxide solution, followed by the addition of morphology-bionic nano-aromatic drugs (50 mg). After stirring for 24 h, the precipitate was obtained by centrifugation (5000 rpm) and washed three times with deionized water. The precipitate was dried at 50 °C for 2 h to remove water molecules from the surface of the nanoparticles. The chemical modification of the bionic plus nano-aromatic drugs was characterized using FT-IR. The encapsulation efficiency of the essential oils was measured by TGA.

### Adhesion of nano-aromatic drugs on wallpaper

The spherical MSNs-based nano-aromatic drugs (10 mg), morphology-bionic nano-aromatic drugs (10 mg), function-bionic nano-aromatic drugs (10 mg) and the bionic plus nano-aromatic drugs (10 mg) were dissolved in 40 mL of deionized water, respectively, and dispersed by ultrasound. Subsequently, wallpaper was added and shaken at room temperature for 3 h. The wallpaper was dried at 50 °C for 2 h to remove moisture. The morphology of the wallpaper treated with the nano-aromatic drugs was characterized by SEM (JSM-6700 F).

### De-adhesion of nano-aromatic drugs on wallpaper

The wallpaper adhered by the nano-aromatic drugs is immersed in water and shaken at room temperature. Samples were taken and dried for 1, 2, 3, and 4 days. The wallpaper morphology after de-adhesion was characterized by SEM (JSM-6700 F).

### Molecular dynamics simulation

Molecular dynamics simulation was performed under constant temperature and pressure and periodic boundary conditions using Gromacs 2018.4 program [32]. The molecular dynamics simulation of three systems of non-bionic nano-aromatic drugs, morphology-bionic nano-aromatic drugs, and function-bionic nano-aromatic drugs based on spherical MSNs was conducted using Charmm36 all-atom force field and TIP3P water model [33], with the addition of 47,579, 71,545, and 85,169 water molecules, respectively. The total atomic number of the system after adding water was 177,203, 355,149, and 307,572. During the molecular dynamics simulation, all the hydrogen bonds involved in the fixed nano-sized aromatic drug system were constrained by the LINCS algorithm [34, 35]. The integration step size was 1 fs. The electrostatic interaction was calculated using the Particle-mesh Ewald (PME) method [36]. The non-bonded interaction cutoff value was set to 10 Å, updated every ten steps. The simulated temperature was controlled to be 300 K by V-rescale [37] temperature coupling method, and the pressure was controlled to be 1 bar by the Parrinello-Rahman method [38]. First, the steepest descent method was used to minimize the energy of the two systems to eliminate the close contact between atoms. A 100 ps NVT balance simulation was then performed at 300 K. Finally, the system was subjected to molecular dynamics simulation for 50 ns, with the conformation preserved once every 10 ps. The visualization of the simulation results was completed using Gromacs embedded program and VMD.

### Animals

The female C57 mice (6 weeks) were purchased from the Academy of Military Medical Sciences of China. CORT was dissolved into saline solution (20 mg/mL) containing 0.1% of DMSO and 0.1% tween-80. The solution of CORT was injected into the mice subcutaneously for 30 days (20 mg·kg^− 1^·day^− 1^). Meanwhile, the mouse cages were pasted with essential oil-treated wallpaper.

### Tail suspension test (TST)

The method of TST was as follows. The mouse’s tail was attached to the tail suspension rope 2 cm from the tip of the tail with a self-adhesive sticker. The mice were then suspended in a tail box. The head of the mouse was about 10 cm from the bottom of the tailstock. The duration of immobility was recorded. The mice were timed for 6 min and the accumulated immobility time was recorded.

### Forced swimming test (FST)

In the last 1 h after the treatment, the mice were put in a transparent cylindrical aquarium. The depth of the water was 5 cm and the temperature was controlled at 24 ℃. The mice stopped struggling in the water or showing a floating state. Only slight body movements could make their heads float on the water, showing a desperate state. After entering the water, the mice were timed for 6 min and adjusted for 2 min, and the accumulated immobility time (s) was recorded within 4 min.

### Novelty suppressed feeding test (NSF)

Mice that were fasted for 24 h were transferred from the feeding area to a particular experimental area (novel environment). The experimental environment was different from the feeding environment, and the light intensity in the experimental environment was greater than that in the feeding environment. In the experiment, the animals were placed outward one by one from one corner into the actual inspection assembly (the actual inspection assembly was a clearing in a fixed area, with the bottom covered with padding, and several foods with regular shapes uniformly placed in the middle). The latency of food intake was recorded. The animal began to chew food, rather than just sniffing or playing with food as the standard, and the latency of food intake was taken as the detection index.

### Open field test

The mice were placed individually in the center of an open field box (48 cm×48 cm), and their spontaneous motor activities were recorded. The following parameters of distance and time to move in the center, periphery, and total area, and the number of times to enter the center area were analyzed by the computer program automatically.

### Immunohistochemistry sections

Heart, liver, spleen, lung, and kidney were removed from the mice, and the tissues were fixed, dehydrated, embedded, and sectioned in formalin solution. The slices were placed into the constant temperature box (60 ℃) for a night and then placed at room temperature for 20 min, and then use xylene solution immerse (10 min), ethanol (volume fraction of 100%, 95%, 85% respectively) soaking gradient (soaking time 3 min and tap water rinse 10 min), wood staining (tap water rinse 10 min), eosin staining (2 min), ethanol solution (volume fraction of 5%, 95%, 100% respectively) gradient dehydration, xylene solution soak (3 min), neutral rubber sealing piece. The seals were observed under a fluorescence microscope, and pictures were taken.

### Statistical analysis

All data were expressed as mean ± SD unless otherwise indicated. Statistical significance was analyzed using one-way ANOVA. Statistical differences in behavioral data were determined using two-way repeated measure ANOVA.

## Results and discussion

### Preparation and characterization of bionic nano-aromatic drugs

Reactive chitosan modified by CYC was synthesized and characterized by FT-IR. As shown in Fig. S1A, there was an obvious characteristic peak of triazine in the spectrum of reactive chitosan, indicating that the chitosan was successfully modified with CYC. Subsequently, MSNRs were prepared and simultaneously encapsulated LEO and BEO, thereby preparing the morphology-bionic nano-aromatic drugs. Furthermore, chitosan and the reactive chitosan were modified on the surface of the morphology-bionic nano-aromatic drugs to prepare the function-bionic nano-aromatic drugs and the bionic plus nano-aromatic drugs, respectively. The surface modifications of function-bionic nano-aromatic drugs and bionic plus nano-aromatic drugs were characterized by FT-IR. As shown in Fig. S1B, the surface of the function-bionic nano-aromatic drugs had significant amino and hydroxyl characteristic peaks, indicating that chitosan was successfully modified on the surface of the morphology-bionic nano-aromatic drugs. Fig. S1C showed the FT-IR of the bionic plus nano-aromatic drugs. The characteristic peak of the triazine group confirmed the preparation of the bionic plus nano-aromatic drugs.

Furthermore, the morphology and internal structure of the bionic nano-aromatic drugs were characterized by SEM and TEM, respectively. As shown in Figure A-D, the morphologies of MSNRs, morphology-bionic nano-aromatic drugs, function-bionic nano-aromatic drugs, and bionic plus nano-aromatic drugs were rod-shaped which observed by SEM. Subsequently, the internal structure of the bionic nano-aromatic drugs was characterized by TEM. The diameter of the bionic nano-aromatic drugs was about 70 nm, and the length was about 170 nm. Before the adsorption of essential oils, the ordered pore structures were observed in MSNRs. After the adsorption of essential oils, the pore structure of morphology-bionic nano-aromatic drugs became less obvious, which might be due to the adsorption of low-contrast essential oils in the pore structure. After the modification of chitosan and reactive chitosan, the surface of the nano-aromatic drugs became rough.

**Fig. 2:**
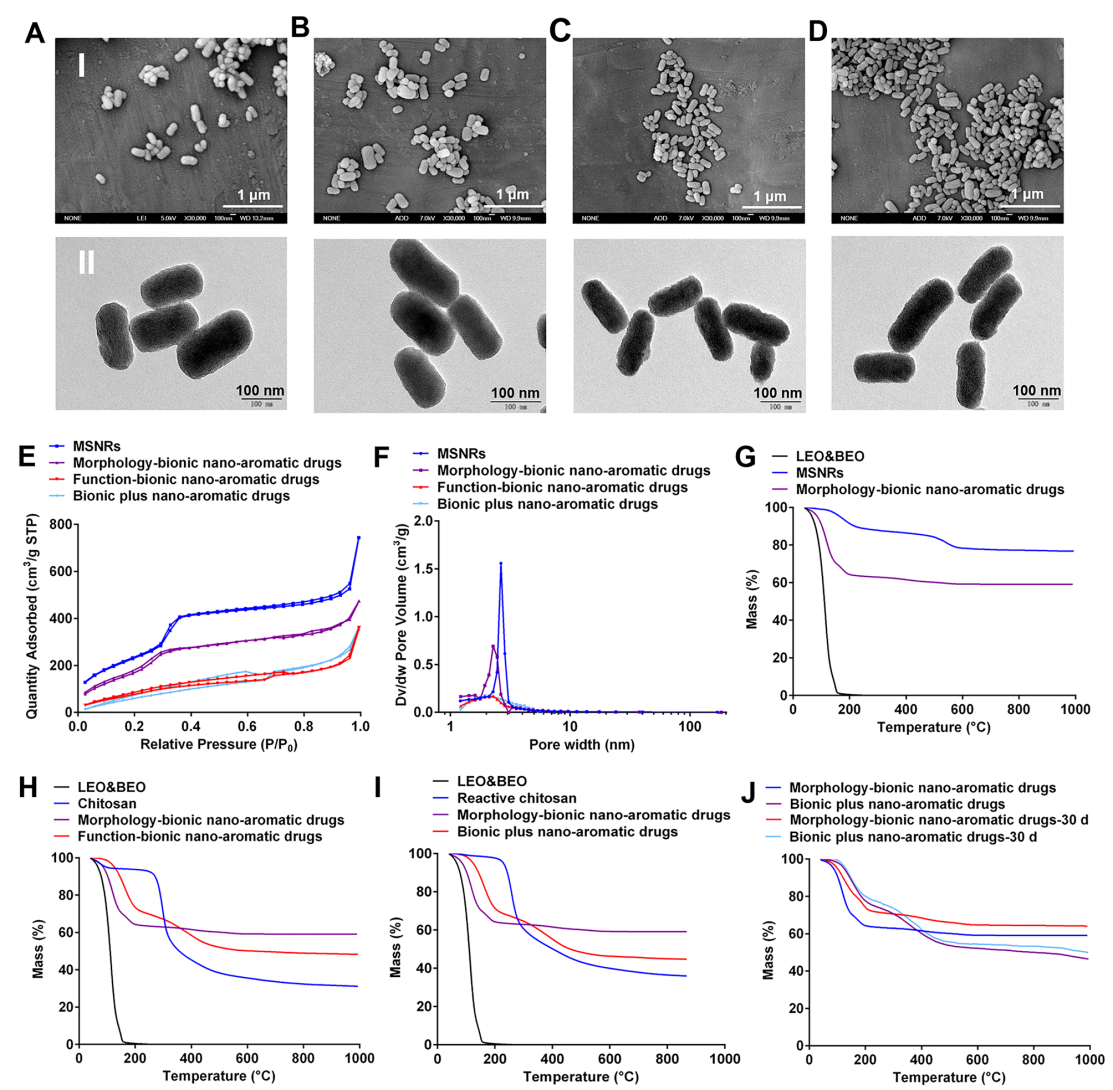
Preparation and characterization of bionic nano-aromatic drugs. (**A**) SEM and TEM images of MSNRs, (**B**) morphology-bionic nano-aromatic drugs, (**C**) functional bionic nano-aromatic drugs, and (**D**) bionic plus nano-aromatic drugs. (I) SEM, (II) TEM (**E**) Nitrogen adsorption-desorption isotherms. (**F**) Pore diameter distributions. (**G**) TGA of morphology-bionic nano-aromatic drugs. (**H**) TGA of function-bionic nano-aromatic drugs. (**I**) TGA of bionic plus nano-aromatic drugs. (**J**) The release of mixed essential oils from nano-aromatic drugs

MSNs encapsulate essential oils through their mesoporous structure. Therefore, the mesoporous performance of MSNs is essential for the encapsulation of essential oils. The nitrogen adsorption-desorption isotherms were used to study the mesoporous structure of the nanoparticles (Fig. 2E). First, the nanoparticles’ specific surface areas and pore volumes were calculated from the data of nitrogen adsorption-desorption isotherms. Before the adsorption of essential oils, the pore volume of MSNRs was 1.83 cm^3^/g, indicating that MSNRs could encapsulate a large number of essential oils. After adsorption of the essential oils, the pore volume of the nano-aromatic drugs decreased to 0.33 cm^3^/g, less than 20% of the pore volume of MSNRs. After the modification of chitosan and reactive chitosan, respectively, the pore volumes of the nano-aromatic drugs were further decreased. Subsequently, the specific surface areas of the nanoparticles were calculated by nitrogen adsorption-desorption isotherms. The specific surface area of MSNRs before adsorption was 752 m^2^/g. This result indicated that MSNRs had a strong adsorption capacity for essential oils. After the essential oils were adsorbed, the specific surface area of the nano-aromatic drugs decreased to 480 m^2^/g. The specific surface area of the nano-aromatic drugs was still large, indicating the performance of the nano-aromatic drugs sustained-release essential oil. Nitrogen adsorption-desorption isotherms calculated the mesoporous diameter distributions of the nanoparticles. As shown in Fig. 2F, MSNRs had a mesoporous structure with a pore diameter of 2.59 nm before the adsorption of essential oils. The pore size distribution of MSNRs was narrow. By contrast, after the adsorption of essential oils, the pore diameter of the nanoparticles decreased slightly, and their distribution became slightly wider. After modification with chitosan and reactivated chitosan, respectively, the pore diameter distributions of the nanoparticles were wide. In particular, the pore diameter distribution peaks of chitosan-modified nano-aromatic drugs and reactive chitosan-modified nano-aromatic drugs were not significant. This result further illustrated that the mesoporous structure of MSNs encapsulated a large number of essential oils.

The encapsulation efficiency of the essential oils in the nano-aromatic drugs was calculated using TGA. As shown in Fig. 2G, the mixture of essential oils of LEO and BEO (LEO&BEO) began thermal decomposition at about 40 ℃ and almost completely decomposed at about 160 ℃. After encapsulation by MSNRs, the decomposition of LEO&BEO began at about 60 ℃ and almost completed at about 190 ℃. This result showed that the encapsulation of MSNRs could improve the thermal stability of essential oils. The encapsulation efficiency of the mixed essential oils in the morphology-bionic nano-aromatic drugs was 27.32%. The encapsulation efficiency of essential oils in the function-bionic nano-aromatic drugs and the bionic plus nano-aromatic drugs was 18.95 and 21.23, respectively (Fig. 2H-I), which was slightly lower than that in the morphology-bionic nano-aromatic drugs. This result might be due to the small number of essential oils escaping from MSNRs during the modification of chitosan and reactive chitosan. Besides, these two kinds of nano-aromatic drugs could also improve the thermal stability of the essential oils. The modification of polymers on the surface of nano-aromatic drugs was expected to improve the sustained release ability of essential oils. As shown in Fig. 2J, after 30 days, 38.35% of the essential oils were released from the morphology-bionic nano-aromatic drugs. By contrast, only 13.96% of the essential oils were released from the bionic plus nano-aromatic drugs. This result indicated that the release rate of essential oils was significantly decreased after MSNRs encapsulated them. The sustained release effect of essential oils was further improved after the reactive chitosan was modified on the surface of nano-aromatic drugs. After above 12 months storage at room temperature, no obvious morphology change was observed, indicating that the nano-aromatic drugs exhibited excellent stability (Fig. S2).

### Adhesion and de-adhesion of nano-aromatic drugs with wallpaper

**Fig. 3:**
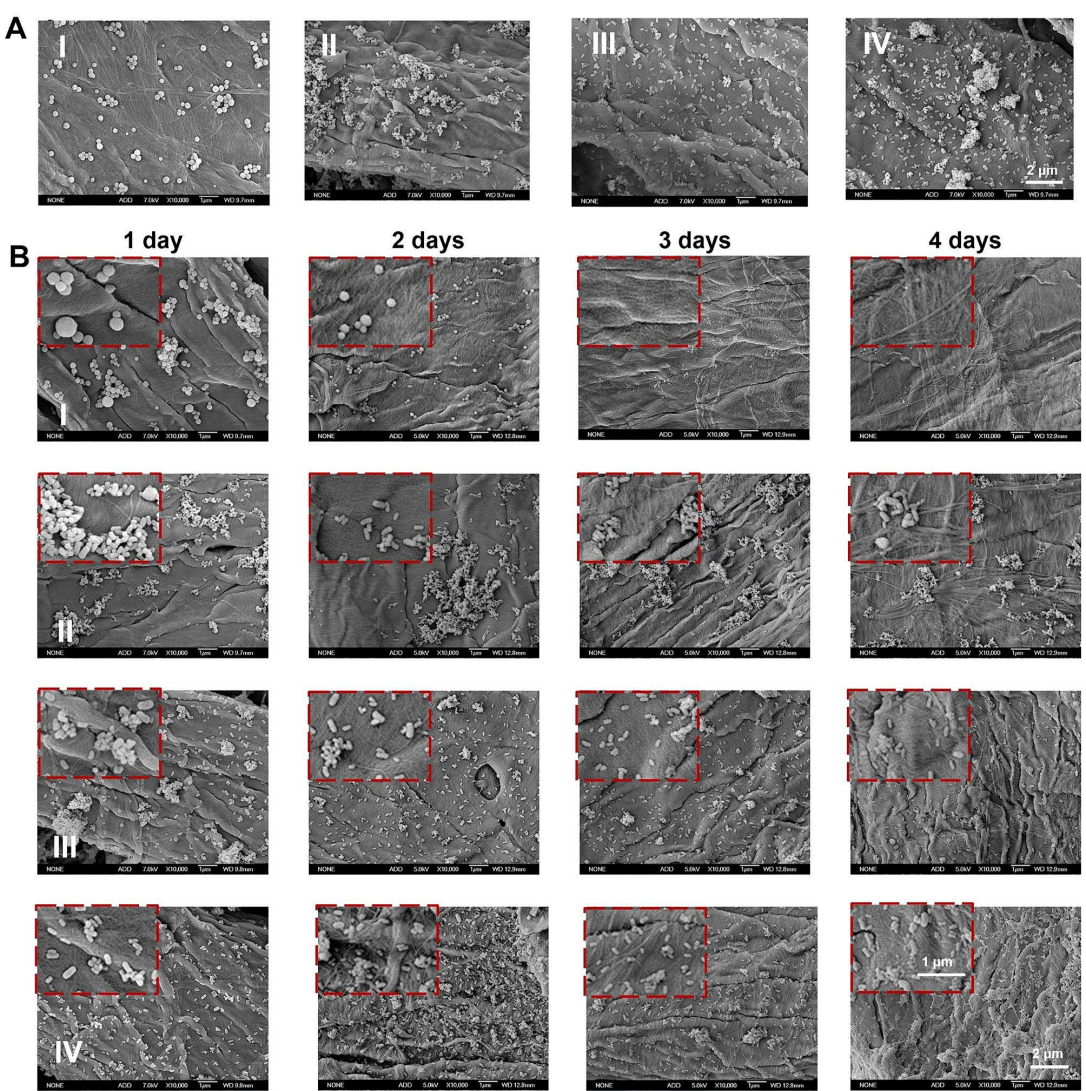
Adhesion and de-adhesion of nano-aromatic drugs with wallpaper. (**A**) Adhesion of nano-aromatic drugs on wallpaper. (**B**) De-adhesion of nano-aromatic drugs from wallpaper. Samples: (I) Non-bionic nano-aromatic drugs. (II) Morphology-bionic nano-aromatic drugs. (III) Function-bionic nano-aromatic drugs. (IV) Bionic plus nano-aromatic drugs

Subsequently, nano-aromatic drugs adhered to wallpaper. As shown in Fig. 3A, the non-bionic spherical nano-aromatic drugs had minimal adhesion to the wallpaper. By contrast, the bionic nano-aromatic drugs had a better adhesion effect on wallpaper. Besides, the morphology-bionic nano-aromatic drugs that adhered to the wallpaper showed aggregation, which might be due to the poor water dispersibility of the morphology-bionic nano-aromatic drugs. By contrast, the wallpaper adhesion abilities of the function-bionic nano-aromatic drugs and the bionic plus nano-aromatic drugs were significantly improved. In particular, compared with the function-bionic nano-aromatic drugs, the wallpaper adhesion ability of the bionic plus nano-aromatic drugs was improved most significantly.

The strong adhesion of nano-aromatic drugs to wallpaper is crucial for the application of nano-aromatic drugs. The de-adhesion of nano-aromatic drugs from wallpaper was then performed. In brief, the wallpaper was immersed in deionized water under stirring. Subsequently, the wallpaper was taken out at a predetermined time, and the retention of the nano-aromatic drugs on the wallpaper was observed using SEM. As shown in Fig. 3B, many non-bionic spherical nano-aromatic drugs had been de-adhered on the first day. On the third day, there were almost no non-bionic nano-aromatic drugs on the wallpaper. All kinds of bionic nano-aromatic drugs were observed on the wallpaper on the fourth day. These results indicated that bionic nano-aromatic drugs could significantly improve their adhesion to wallpaper. Among all bionic nano-aromatic drugs, the release rate of morphology-bionic nano-aromatic drugs was the fastest. On the fourth day, only a tiny amount of morphology-bionic nano-aromatic drugs were observed on the wallpaper. In contrast, only a tiny amount of function-bionic nano-aromatic drugs were de-adhered from the wallpaper on the fourth day, indicating that the polysaccharide modification significantly improved the adhesion ability of the bionic nano-aromatic drugs. Moreover, the bionic plus nano-aromatic drugs, which were endowed with the covalent bond between the nano-aromatic drugs and the wallpaper based on the function-bionic nano-aromatic drugs, hardly showed the de-adhesion on the fourth day. These results indicated that the covalent bonds based on the function-bionic nano-aromatic drugs could significantly improve the adhesion ability of the nano-aromatic drugs on wallpaper.

### Molecular dynamics simulation between bionic nano-aromatic drugs and wallpaper

The main ingredient of wallpaper is cellulose. To simplify the simulation conditions, we simplified the wallpaper to cellulose and reduced the size of the nano-aromatic drugs. MSNRs were simplified as SiO_2_ for molecular dynamics simulation. The crystal structure of SiO_2_ was obtained and cut to obtain a cuboid SiO_2_ structure. SiO_2_ spheres with a diameter of 10 nm (non-bionic nano-aromatic drugs) and rod-like SiO_2_ with a diameter of 8 nm and a length of 12 nm (morphology-bionic nano-aromatic drugs) were constructed using VMD, respectively, as shown in Fig. S3A and S3B. In addition, chitosan molecules were modified on the surface of rod-like SiO_2_ (function-bionic nano-aromatic drugs). The chitosan molecule was composed of 15 units attached to the Si atom, as shown in Fig. S3C and S3D. The cellulose structure was downloaded from the Cambridge Crystallographic Data Centre (CCDC) and shown in Fig. S4. We analyzed the complex structure after molecular dynamics simulation, as shown in Fig. S5. Cellulose could interact with non-bionic nano-aromatic drugs, morphology-bionic nano-aromatic drugs, and function-bionic nano-aromatic drugs.

**Fig. 4:**
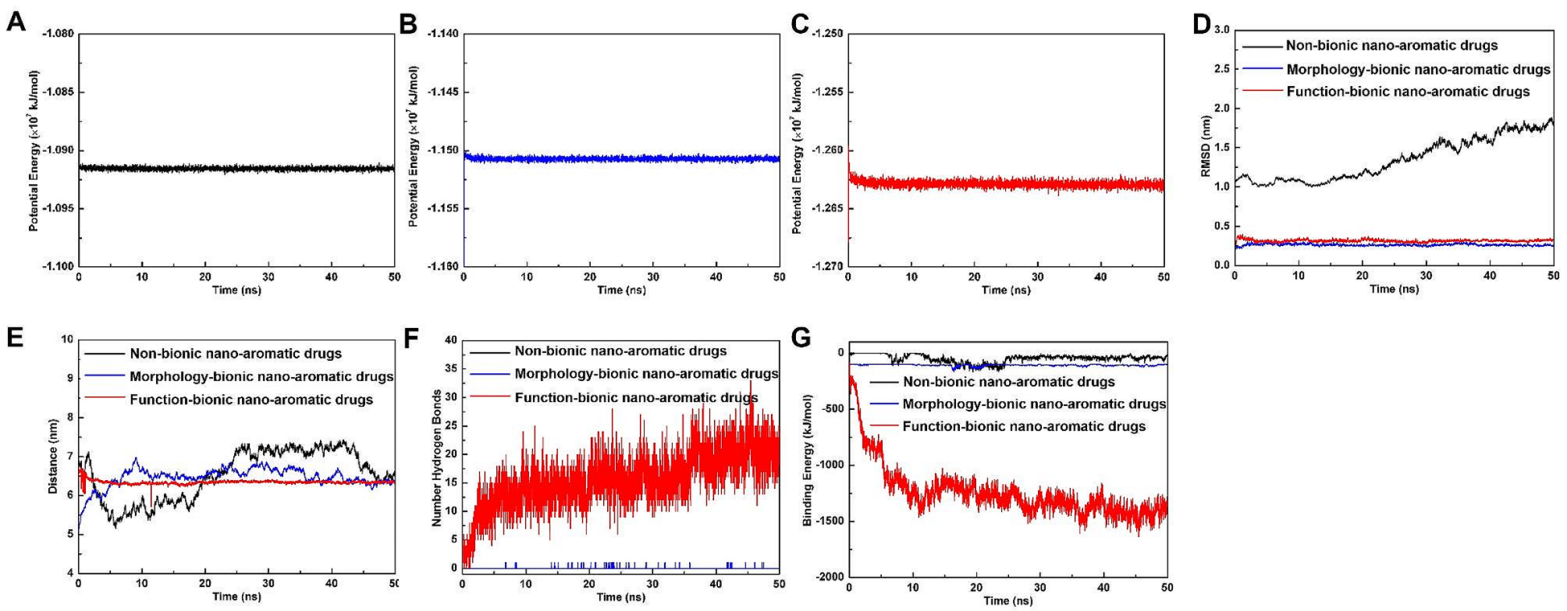
Molecular dynamics simulation between bionic nano-aromatic drugs and wallpaper. (**A**) Variation of system potential energy with molecular dynamics simulation time of non-bionic nano-aromatic drugs, (**B**) morphology-bionic nano-aromatic drugs and (**C**) function-bionic nano-aromatic drugs. (**D**) Variation of RMSD of cellulose in the system with molecular dynamics simulation time. (**E**) Variation of the mass center distance and (**F**) variation of hydrogen bond number between cellulose and nano-aromatic drugs were investigated. (**G**) Variation of the binding energy of cellulose and nano-aromatic drugs with time

The variation of system potential energy is an important basis to judge whether the whole system is stable. Figure 4A-C showed the variation of the system potential energy with time during the molecular dynamics simulation. There was no significant fluctuation of the system potential energy during the simulation. The average values were (-1.09 ± 0.001) × 107 kJ/mol, (-1.15 ± 0.001) × 107 kJ/mol and (-1.26 ± 0.001) × 107 kJ/mol, respectively, with the fluctuation amplitude of only 0.09%. The molecular dynamics simulation process was stable and reliable.

The dynamic of the system in the water environment can be approximately expressed by the root mean square deviation (RMSD). RMSD can describe the degree of difference between the conformation of the system at a specific moment and the target conformation and is an essential index for judging whether the simulation system converges or not. The change of RMSD of cellulose atoms with time was shown in Fig. 4D. In the non-bionic nano-aromatic drugs, the fluctuation amplitude of cellulose was more significant than that of the morphology and function-bionic nano-aromatic drugs. The average values of RMSD of the three systems were 1.715 ± 0.078 nm, 0.264 ± 0.011 nm, and 0.316 ± 0.013 nm, respectively, indicating that the combination of cellulose was not as stable as that of the morphology-bionic nano-aromatic drugs and the function-bionic nano-aromatic drugs on the surface of the non-bionic nano-aromatic drugs. According to the combination mode, it could be speculated that the main reason was that the contact area between the non-bionic nano-aromatic drugs and cellulose was relatively small, and the interaction between them was relatively weak. These reasons led to the difficulty of cellulose in achieving a stable state on the non-bionic nano-aromatic drugs.

To further study cellulose-binding on the surface of nano-aromatic drugs, we analyzed the changes in the distance between the mass centers and the number of hydrogen bonds during molecular dynamics simulation. As shown in Fig. 4E, in the non-bionic nano-aromatic drugs, the distance between cellulose and the mass center of the nano-aromatic drugs fluctuated significantly in the simulation process, and it firstly decreased to about 5.5 nm, then increased to about 7.5 nm after 30 ns, and gradually decreased to 6.5 nm after 45 ns. In the morphology-bionic nano-aromatic drugs, the mass center distance between cellulose and the nano-aromatic drugs fluctuated within the range of 6–7 nm, and no significant change was observed. In the function-bionic nano-aromatic drugs, the distance between cellulose and the mass center of the nano-aromatic drugs was relatively more stable in the simulation process, with an average value of 6.34 nm. By analyzing the changes in the distance between the mass centers of cellulose and these three systems in the simulation process, we could find that the distance between the mass centers of cellulose and the nano-aromatic drugs fluctuated the most in the non-bionic nano-aromatic drugs, indicating that their combination stability was the worst. The morphology-bionic nano-aromatic drugs and the function-bionic nano-aromatic drugs were more stable than non-bionic nano-aromatic drugs. The combination stability of these three systems and cellulose was in the order of function-bionic nano-aromatic drugs > morphology-bionic nano-aromatic drugs > non-bionic nano-aromatic drugs.

In the function-bionic nano-aromatic drugs, the surface of the nano-aromatic drugs was modified with chitosan molecule, which contained a large number of hydroxyl groups that could be combined with cellulose as hydrogen bond donor and acceptor. Therefore, to further study the interaction between cellulose and the nano-aromatic drugs, Fig. 4F explained the number of hydrogen bonds between cellulose and these three nano-aromatic drugs, respectively. In the non-bionic nano-aromatic drugs and the morphology-bionic nano-aromatic drugs, there was almost no hydrogen bond between cellulose and the nano-aromatic drugs. While in the functional bionic nano-aromatic drugs with chitosan modification, the hydrogen bond between cellulose and chitosan was gradually increased and stabilized at about 20. This result indicated that chitosan could form more hydrogen bonds with cellulose, enhancing the interaction between cellulose and the function-bionic nano-aromatic drugs.

To study the affinity of cellulose and nano-aromatic drugs, we analyzed the changes in the binding energy of cellulose and nano-aromatic drugs during the molecular dynamics simulation, as shown in Fig. 4G. The binding energies of cellulose to the non-bionic nano-aromatic drugs and the morphology-bionic nano-aromatic drugs were relatively small, with the average values of -40.89 ± 13.90 kJ/mol and  -109.04 ± 5.00 kJ/mol, respectively. The binding energy of the function-bionic nano-aromatic drugs with the surface-modified chitosan to cellulose was the strongest, with an average value of -1419.95 ± 62.54 kJ/mol.

### The binding mode of wallpaper and nano-aromatic drugs

**Fig. 5:**
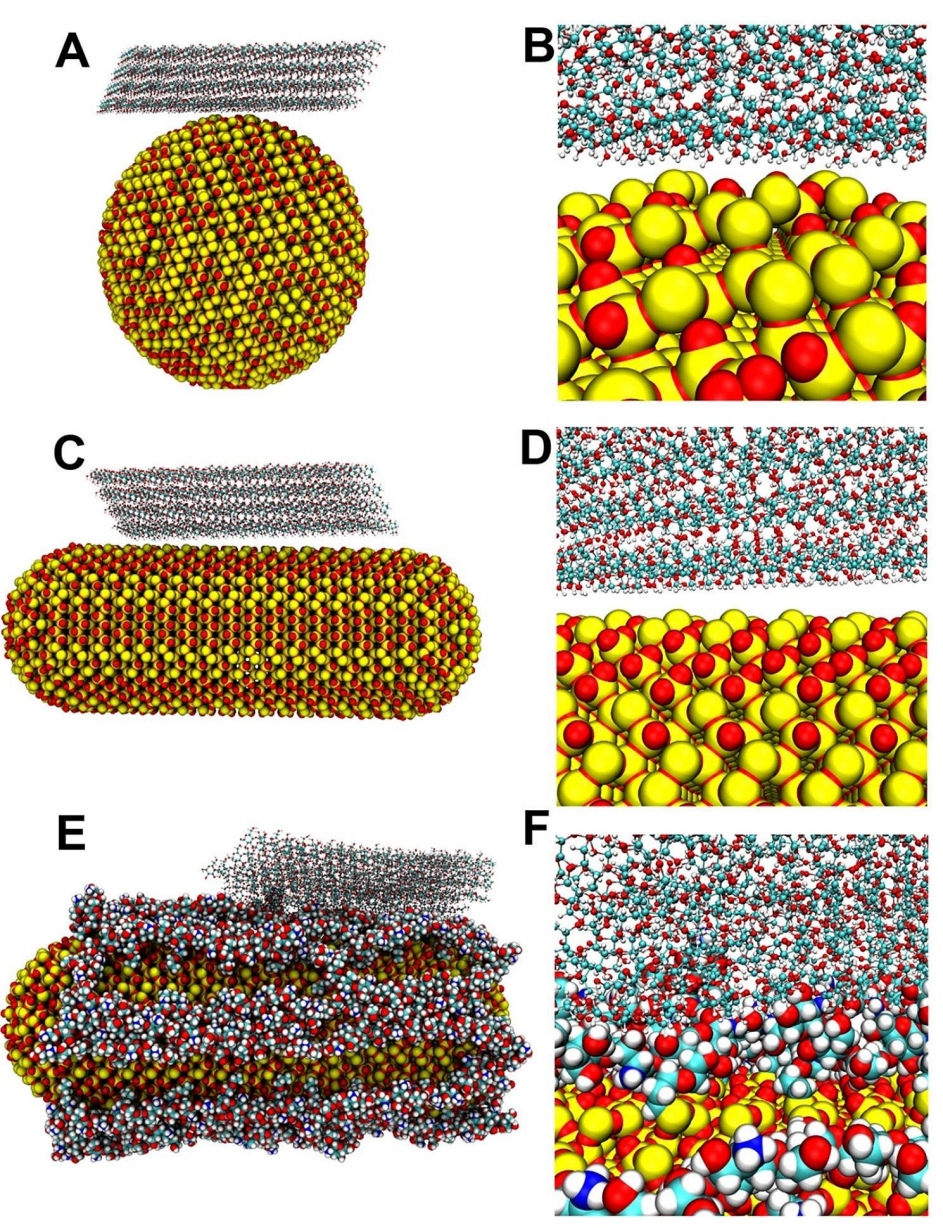
Modes of cellulose-binding on the surface of nano-aromatic drugs. (**A-B**) The combination mode of cellulose and non-bionic nano-aromatic drugs. (**C-D**) The combination mode of cellulose and morphology-bionic nano-aromatic drugs. (**E-F**) The combination mode of cellulose and functional bionic nano-aromatic drugs. Si, O, C, N, and H atoms were represented by yellow, red, cyan, blue, and white, respectively

To further study the binding mode of wallpaper and nano-aromatic drugs, the binding mode of cellulose on the surface of nano-aromatic drugs after molecular dynamics simulation was shown in Fig. 5. The cellulose combination modes with the non-bionic nano-aromatic drugs and the morphology-bionic nano-aromatic drugs were mainly that the hydroxyl of the cellulose faces the surface of the nano-aromatic drugs. However, as the Si atoms in the non-bionic aromatic drugs and the morphology-bionic nano-aromatic drugs were larger in volume than the O atoms and had a specific positive charge, the hydroxyl of the cellulose was difficult to directly contact with the O atoms of the non-bionic aromatic drugs and the morphology-bionic nano-aromatic drugs. Therefore, it was difficult for cellulose to form hydrogen bonds with non-bionic nano-aromatic drugs and morphology-bionic nano-aromatic drugs. The spatial structure of cellulose had not changed significantly after 50 ns molecular dynamics simulation. After chitosan was modified on the surface of nano-aromatic drugs, cellulose and chitosan molecules form strong interactions to change the cellulose’s structures. The cellulose was embedded between the chitosan on the surface of the function-bionic nano-aromatic drugs so that a large number of hydrogen bonds and hydrophobic interaction could be formed. The interaction and affinity between the cellulose and the function-bionic nano-aromatic drugs were significantly enhanced.

In summary, to investigate the adhesion of nano-aromatic drugs to the wallpaper surface, the molecular dynamics simulation was used to study the binding mode of cellulose on the surface of nano-aromatic drugs. By analyzing the changes of convergence parameters in the molecular dynamics simulation process, it was found that the cellulose reached a stable state on the surface of the nano aromatic drugs. The analysis of hydrogen bonds and binding energy found that almost no hydrogen bond was formed between cellulose and non-bionic nano-aromatic drugs or morphology-bionic nano-aromatic drugs. However, a large number of hydrogen bond interactions were formed with the surface of function-bionic nano-aromatic drugs. Finally, by analyzing the combination mode of cellulose and the nano-aromatic drugs after molecular dynamics simulation, it was found that after cellulose was combined on the surfaces of non-bionic nano-aromatic drugs and morphology-bionic nano-aromatic drugs, the spatial structure did not change significantly. While after cellulose was combined with the function-bionic nano-aromatic drugs, more hydrogen bonds could be formed. The spatial structure of cellulose could be affected to better combine with chitosan on the surface of the nano-aromatic drugs. The molecular simulation results were consistent with the experimental results, which proved that the surface modification of chitosan with nano-aromatic drugs could enhance the affinity of cellulose with nano-aromatic drugs to a certain extent and provided theoretical guidance for the subsequent research.

### The behavior effects of nano-aromatic drugs on the prevention of depression

We used a continuous subcutaneous injection of CORT for 30 days to create external stress stimulation and induce depression model mice [39–43]. In the external stimulation process, the wallpaper adhered with the bionic plus nano-aromatic drugs was pasted on the wall of the mouse cage to relieve stress, thereby achieving the effect of preventing depression. Biological safety is the prerequisite for the application of nano-aromatic drugs. We evaluated the damage of nano-aromatic drugs to the principal organs of mice by HE staining. As shown in Fig. S6, continuous injection of CORT did not cause damage to the principal organs such as the heart, liver, spleen, lung, and kidney. Besides, no toxicity was found in organs of the mice treated with the free aromatic drugs or the nano-aromatic drugs. These results indicated that both the free aromatic drugs and the nano-aromatic drugs have excellent biosafety.

**Fig. 6:**
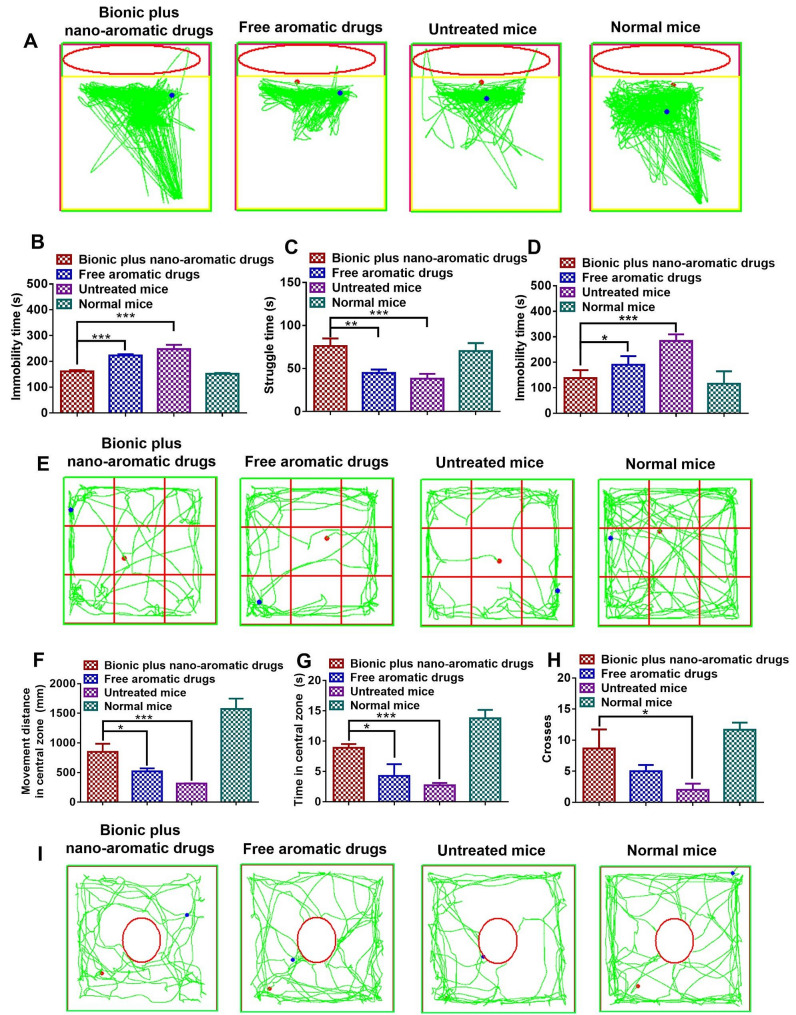
Evaluation of despair and exploratory behavior in mice. (**A**) The movement routes of mice in containers of FST. (**B**) Immobility time of mice in FST. (**C**) Struggling time of mice in FST. (**D**) Immobility time of mice in TST. (**E**) The movement routes of mice in open field tests. (**F**) Movement distances of mice in the central zone. (**G**) Time in the central zone of mice. (**H**) Number to enter the central zone of mice. (**I**) The movement routes of mice in NSF tests. The mean ± SD was shown (*n* = 3). *P < 0.05, **P < 0.01, ***P < 0.005

Subsequently, we evaluated the efficacy of nano-aromatherapy in the prevention of depression. First, we evaluated the desperate state of the mice by forced swimming test (FST) and tail suspension test (TST) [44]. FST was a behavioral experiment for evaluating the desperate state of mice [45–48]. FST involved placing mice in a cylindrical container filled with water. The mice could not escape from the container and were forced to swim. The desperate state of the mice was evaluated by observing the struggling time of the mice and the immobility time when the mice stopped trying to struggle and failed to move. Figure 6A showed the swimming routes of the mice inside the container. Figure 6B and C quantified immobility time and struggling time, respectively. The results showed that after induction by CORT, the immobility time of mice was significantly increased, and the struggling time was significantly reduced, showing a significant state of despair. However, after treatment with free aromatic drugs, the desperate state of mice did not improve significantly. By contrast, after treatment with nano-aromatic drugs, the desperate state of mice was significantly improved. Moreover, the struggling time of mice after treatment with nano-aromatic drugs was even slightly longer than that of normal mice. This result indicated that the nano-aromatic drugs could significantly alleviate the desperate state of mice under a stressful environment.

TST created an inescapable state by suspending the mouse tail and then observing the immobility time of mice [49–52]. As shown in Fig. 6D, the immobility time was significantly increased in mice after injection of CORT. This result indicated that CORT could induce severe despair in mice. However, after treatment with nano-aromatic drugs and free aromatic drugs, the immobility time of mice was significantly reduced. In particular, the immobility time of mice treated with nano-aromatic drugs was close to that of normal mice. The FST and TST results together indicated that aromatherapy could relieve despair, and the effect of nano-aromatic drugs was better than free aromatic drugs.

Furthermore, we tested the exploratory behavior of mice by open field test and novelty suppressed feeding test (NSF). Open field test is a behavioral detection method for exploring and spontaneous movement [53–55]. Mice with severe depression are afraid of the open field, so they tend to move along the edge of the open field. Figure 6E showed the movement route of mice in the open field. Figure 6F-H respectively quantified the moving distance in the central zone, moving time in the central zone, and times of entering the central zone of mice. The results showed that the movement distance, movement time, and times of entering the central zone of depression mice induced by CORT decreased significantly. This result indicated that CORT significantly reduced the spontaneous behavior of mice based on exploration. After treatment with aromatic drugs, especially nano-aromatic drugs, the movement distance, movement time, and times of entering the central zone of mice were significantly increased. However, there was still a gap with normal mice. This result showed that nano-aromatic drugs could significantly alleviate the weakening of exploratory behavior in mice under a stressful environment, thus having a positive effect of preventing depression.

NSF is to put fasting mice in a novel environment [56–58]. Depressed mice are afraid of the novel environment and then fear or hesitate to eat. Therefore, the exploratory behavior of mice in the novel environment can be evaluated through the latency to feed, and then the depression state of mice can be studied. Figure 6I showed the movement route of mice in the novel environment. Fig. S7 quantified the mice’s latency to feed. The results showed that the latency of depression mice induced by CORT was significantly increased. After cultured with aromatic drugs, the latency of mice decreased significantly. Especially, the mice’s latency to feed was significantly reduced after treatment with nano-aromatic drugs.

### The mechanism exploration of nano-aromatic drugs preventing depression

**Fig. 7:**
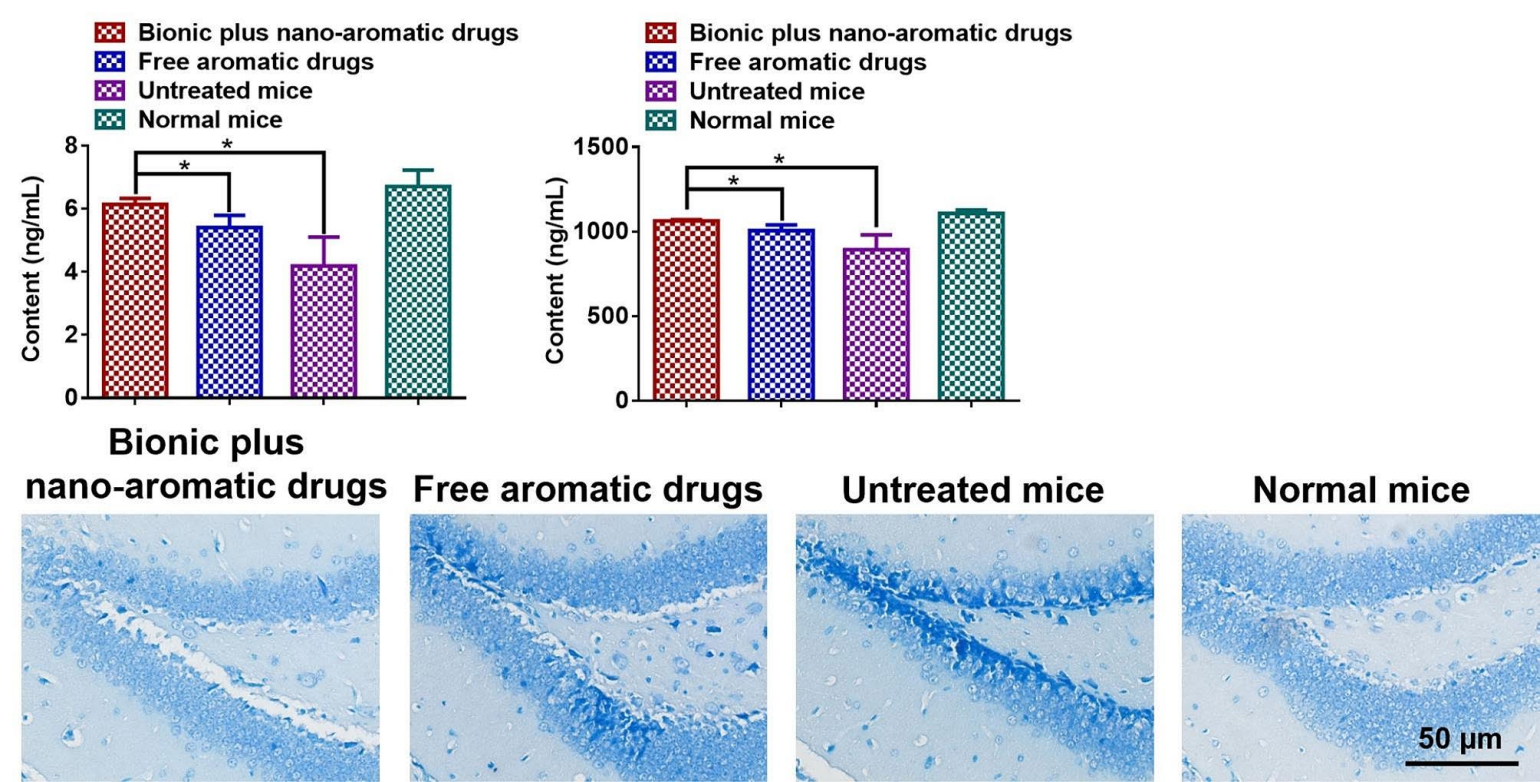
The mechanism exploration of nano-aromatic drugs preventing depression. (**A**) The content of 5-HT in the brain. (**B**) The content of NE in the brain. (**C**) Nissl staining of the hippocampal dentate gyrus in mice. The mean ± SD was shown (*n* = 3). *P < 0.05

Monoamine neurotransmitters, such as 5-HT and NE, are closely related to depression [14, 59–62]. Therefore, we measured the contents of 5-HT and NE in the brain of the treated mice. As shown in Fig. 7A, the 5-HT content in the brain of CORT-induced depression mice was significantly reduced compared with normal mice. After treatment with the mixed essential oils, the content of 5-HT in the brain of the mice was increased. In particular, the content of 5-HT was significantly increased after treatment with nano-aromatic drugs. Figure 7B showed the amount of NE in the mouse brains. Subcutaneous injection of CORT significantly reduced NE content. The subcutaneous injection of CORT in mice was accompanied by a significant increase in NE content via nano-aromatic drug treatment. These results indicated that the nano-aromatic drugs could significantly alleviate the reduction of monoamine neurotransmitters in the stressful environment.

Subsequently, Nissl bodies in the mouse hippocampus were analyzed by Nissl staining. As shown in Fig. 7C, after CORT stressful stimulation, the structure of Nissl bodies in the mouse dentate gyrus was severely damaged. After treatment with the free essential oils, a large number of damaged Nissl bodies were still observed. By contrast, after treatment with nano-aromatic drugs, the Nissl bodies in the dentate gyrus of mice were significantly improved. These results indicated that the nano-aromatic drugs had a noticeable neuroprotective effect.

## Conclusion

In summary, we prepared bionic nano-aromatic drugs encapsulating a blend of essential oils to prevent depression. First, inspired by the fact that wallpaper is prone to mold, we have successively prepared morphology-bionic nano-aromatic drugs, function-bionic nano-aromatic drugs, and bionic plus nano-aromatic drugs. We have found that the bionic nano-aromatic drugs significantly improve nanoparticles’ adhesion effect on wallpaper through wallpaper adhesion and desorption experiments. Molecular dynamics simulation showed that the morphology-bionic nano-aromatic drugs could improve the adhesion of nano-aromatic drugs to wallpaper by increasing their adhesion area. Based on the morphology-bionic nano-aromatic drugs, the functional-bionic nano-aromatic drugs formed many hydrogen bonds with cellulose on the wallpaper, induced the cellulose to change the spatial structure. They were embedded among chitosan molecules of the function-bionic nano-aromatic drugs so that the adhesion of the nano-aromatic drugs on the wallpaper was remarkably improved through the hydrogen bonds and the hydrophobic interaction. Moreover, the bionic plus nano-aromatic drugs introduced covalent bonds based on the function-bionic nano aromatic drugs so that the adhesion of the bionic nano-aromatic drugs on the wallpaper was further improved. Subsequently, we explored the efficacy of bionic plus nano-aromatic drugs-treated wallpaper in preventing depression. The depression model mice were constructed under chronic stress for a long time through subcutaneous injection of CORT. In a stressful environment, the nano-aromatic drugs could significantly alleviate the desperate state of mice and improve their exploratory behavior, thereby relieving CORT-induced depressive state. These positive effects might be since the nano-aromatic drugs could increase the content of monoamine neurotransmitters and had a neuroprotective effect. Therefore, the bionic plus nano-aromatic drugs had excellent application prospects.

## Electronic supplementary material

Below is the link to the electronic supplementary material.

Supplementary Material 1

## Data Availability

No datasets were generated or analysed during the current study.
