# Supplementary material for: Microbial bionic nano-aromatic drugs for prevention of depression induced by chronic stress
Source: J Nanobiotechnology. 2024 Apr 12;22:173. doi: 10.1186/s12951-024-02382-y (PMC11015588; doi:10.1186/s12951-024-02382-y)
Supplement: Supplementary file 1 — Supplementary Material 1 [file 12951_2024_2382_MOESM1_ESM.docx]

**Supporting Information**

**Title**

**Microbial bionic nano-aromatic drugs for prevention of depression induced by chronic stress**

*Ruiyuan Liu^1†^, Tianlu Zhang^2,6†^, Chaobo Bai^3^, Jing Chen^3^, Xin Zhang^2,6^, Guiying Liu^5*^, Songjie Shen^4*^, Junliang Yuan^3*^, Zhiguo Lu^2,6*^*

^1^College of Pharmacy, Heze University, Heze, 274015, PR China

^2^State Key Laboratory of Biochemical Engineering, Institute of Process Engineering, Chinese Academy of Sciences, Beijing, 100190, PR China

^3^Department of Neurology, Peking University Sixth Hospital, Peking University Institute of Mental Health, Beijing, 100191, PR China

^4^Department of Breast Surgery, Peking Union Medical College Hospital, Peking Union Medical College, Chinese Academy of Medical Sciences, Beijing 100730, PR China

^5^Department of Pediatrics, Capital Medical University Affiliated Beijing Anzhen Hospital, Beijing, 100029, PR China

^6^Key Laboratory of Biopharmaceutical Preparation and Delivery, Institute of Process Engineering, Chinese Academy of Sciences, Beijing, 100190, PR China

^∗^Corresponding authors.

E-mail addresses: liugvying@126.com (G. Liu), [shensj@pumch.cn](mailto:shensj@pumch.cn) (S. Shen), junliangyuan@bjmu.edu.cn (J. Yuan), zglu18@ipe.ac.cn (Z. Lu)

^†^These authors contributed equally to this work.


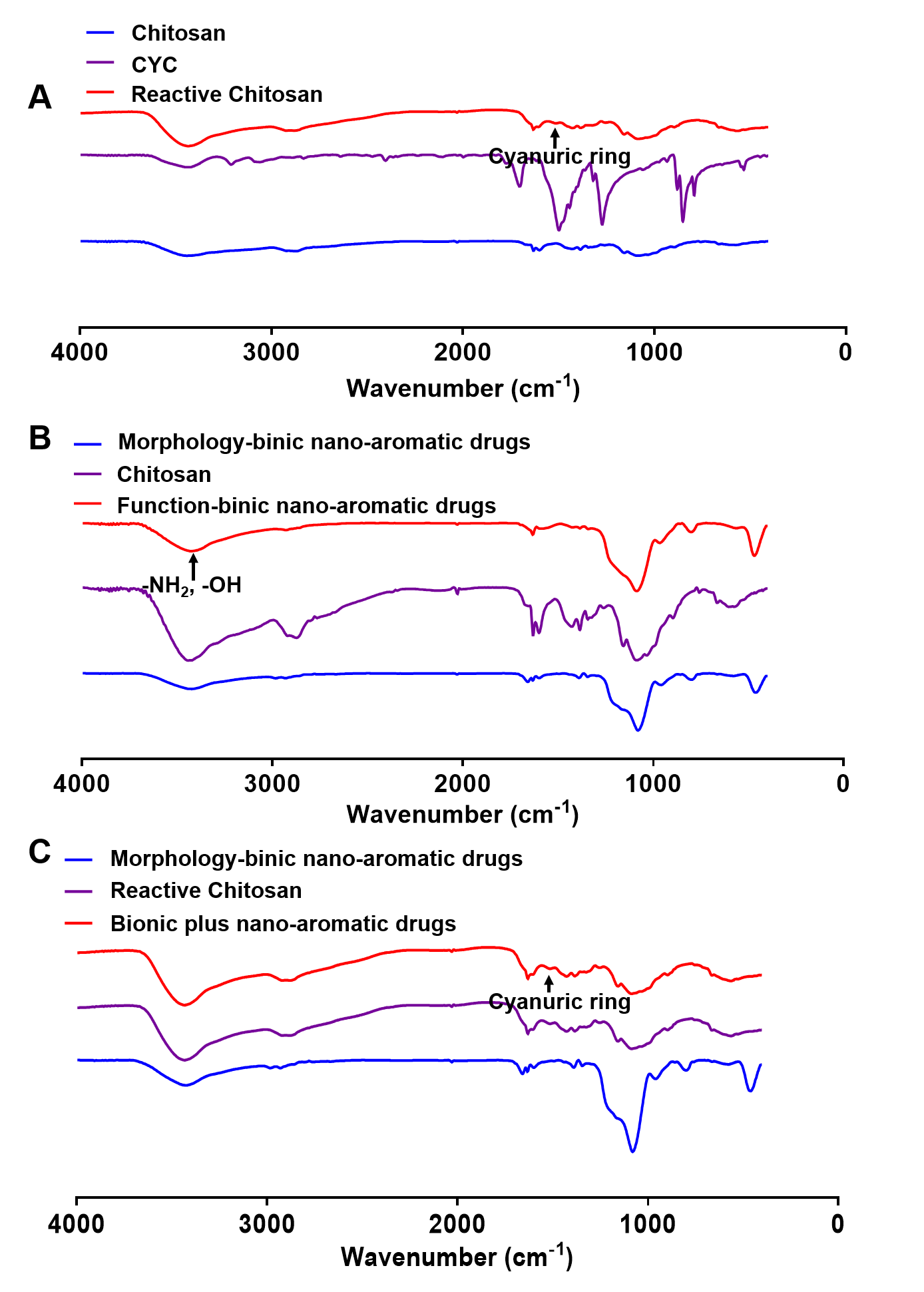


**Fig. S1** The FT-IT spectra. (**A**) The FT-IR spectra of reactive chitosan. (**B**) The FT-IR spectra of function-bionic nano-aromatic drugs. (**C**) The FT-IR spectra of bionic plus nano-aromatic drugs.


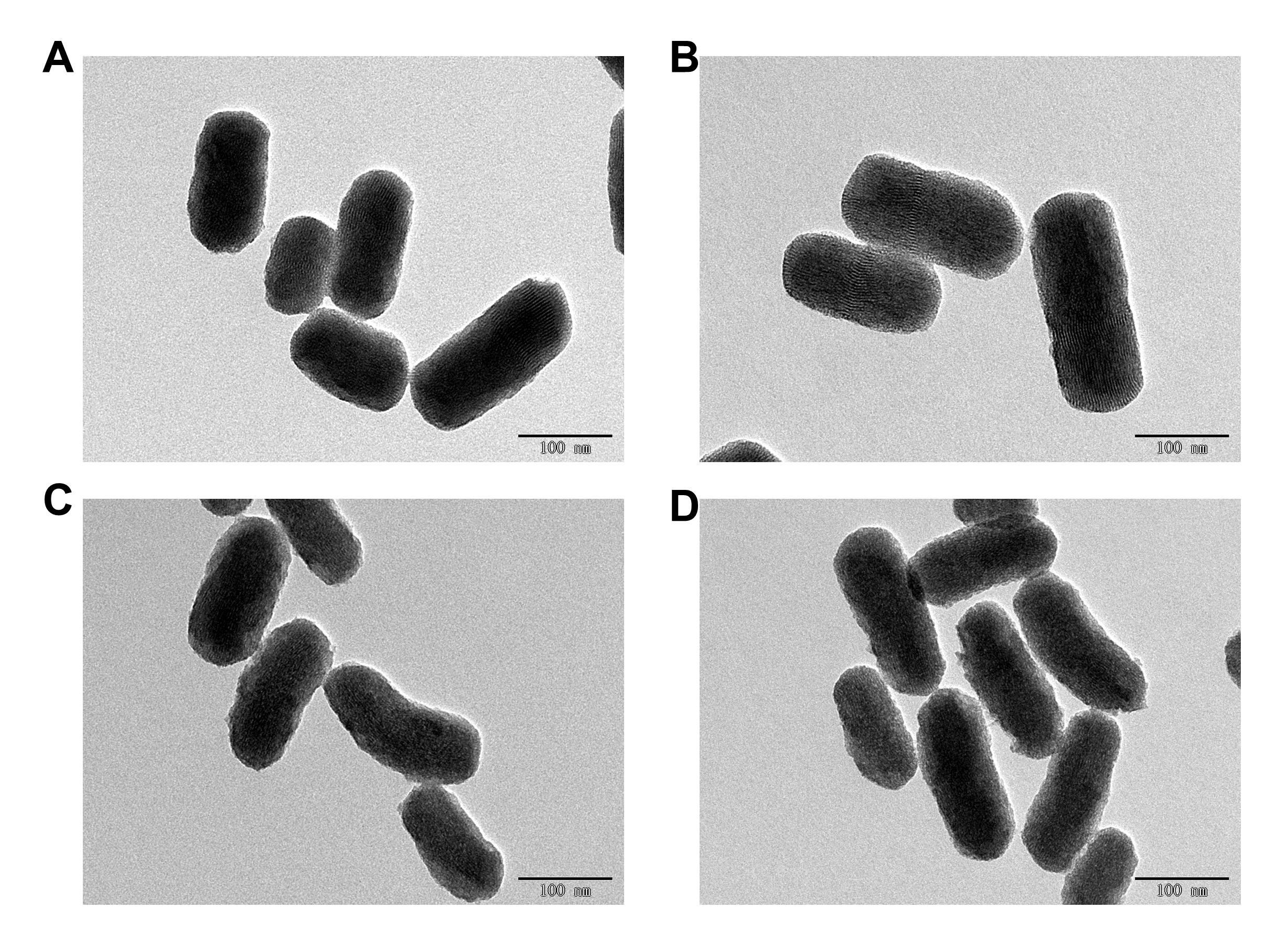


**Fig. S2** Stability of the nano-aromatic drugs. (A) MSNRs. (B) morphology-bionic nano-aromatic drugs. (C) functional bionic nano-aromatic drugs. (D) bionic plus nano-aromatic drugs. Scale bar: 100 nm.


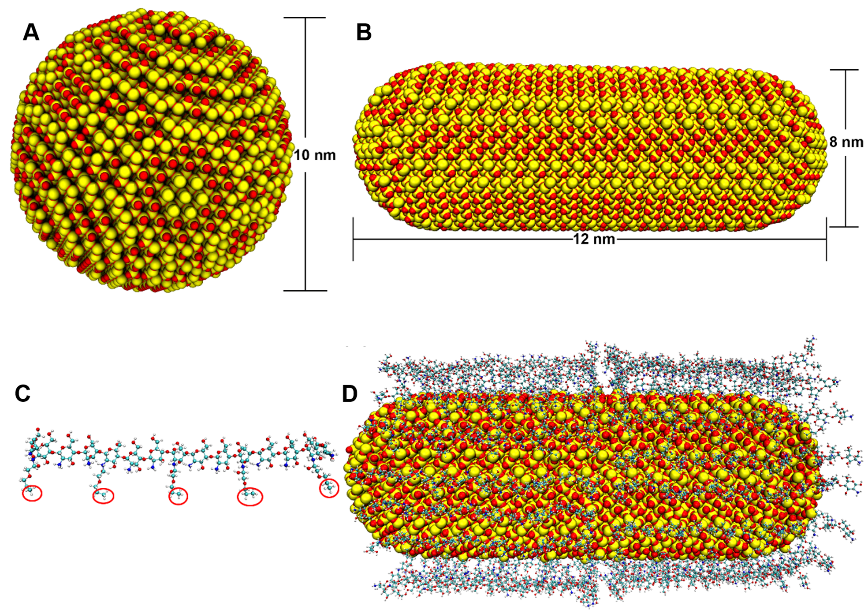


**Fig. S3** Structure diagrams of nano-aromatic drugs model. (**A**) Non-bionic nano-aromatic drugs. (**B**) Morphology-bionic nano-aromatic drugs. (**C**) Chitosan. (D) Function-bionic nano-aromatic drugs.


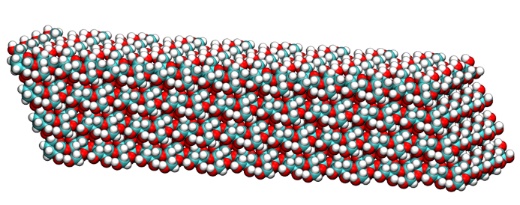


**Fig. S4** Structure diagrams of cellulose model.


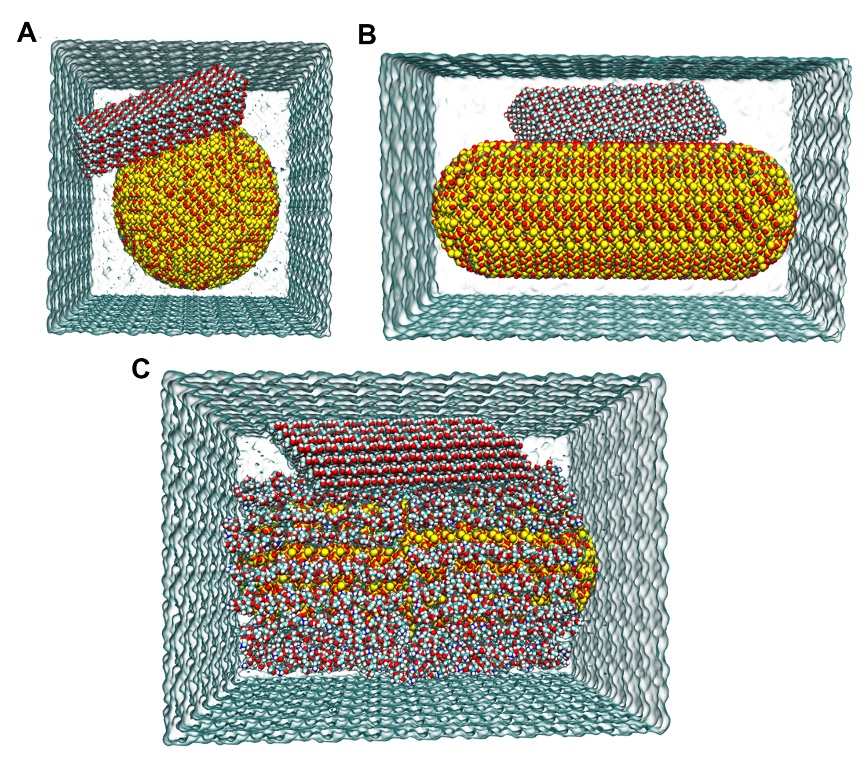


**Fig. S5** The results of molecular dynamics. (**A**) Non-bionic nano-aromatic drugs and cellulose. (**B**) Morphology-bionic nano-aromatic drugs and cellulose. (**C**) Function-bionic nano-aromatic drugs and cellulose.


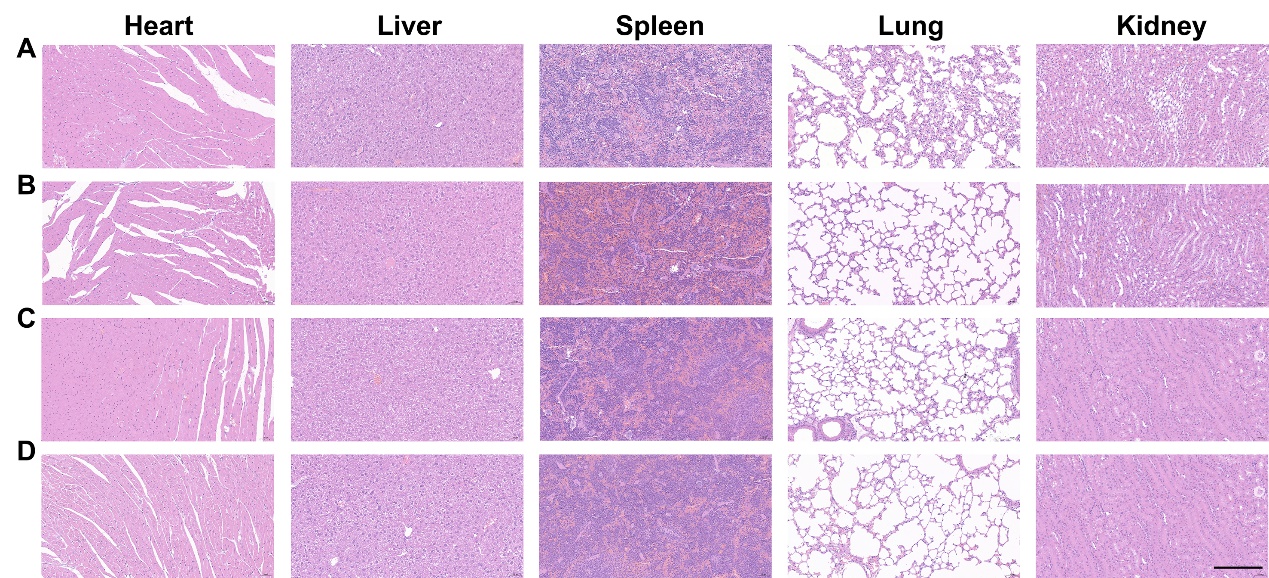


**Fig. S6** HE staining of hearts，livers，spleens，lungs，kidneys. (**A**) Bionic plus nano-aromatic drugs. (**B**) Free aromatic drugs. (**C**) Untreated mice. (**D**) Normal mice. Scale bar: 200 μm.

**Fig. S7** Quantification of the mice’s latency to feed.
